# Photosynthetic Traits of Páramo Plants Subjected to Short-Term Warming in OTC Chambers

**DOI:** 10.3390/plants11223110

**Published:** 2022-11-15

**Authors:** María Elena Solarte, Yisela Solarte Erazo, Elizabeth Ramírez Cupacán, Camila Enríquez Paz, Luz Marina Melgarejo, Eloisa Lasso, Jaume Flexas, Javier Gulias

**Affiliations:** 1 Laboratorio de Ecofisiología Vegetal, Grupo de Investigación Biología de Páramos y Ecosistemas Andinos, Departamento de Biología, Facultad de Ciencias Exactas y Naturales, Universidad de Nariño, Pasto 52001, Colombia; 2 Laboratorio de Fisiología y Bioquímica Vegetal, Departamento de Biología, Facultad de Ciencias, Universidad Nacional de Colombia-Sede Bogotá, Bogota 111321, Colombia; 3 Grupo de Ecología y Fisiología Vegetal EcoFiv, Departamento de Ciencias Biológicas, Universidad de los Andes, Bogota 111711, Colombia; 4 Grupo de Investigación en Biología Vegetal en Condiciones Mediterráneas, Departamento de Biología, Universitat de Les Illes Balears (UIB), 07122 Palma, Spain

**Keywords:** páramo, climate change, land use history, photosynthesis, open top chamber OTC

## Abstract

Global warming and changes in land use are some of the main threats to high mountain species. Both can interact in ways not yet assessed. In this study, we evaluated the photosynthetic responses of six common páramo species within a warming experiment using open-top chambers (OTC) in conserved páramo areas with different land use histories. We did not find significant differences in the photochemical performance of the species as measured through Fv/Fm, ETR, and NPQ in response to passive warming, indicating that warmed plants are not stressed. However, NPQ values were higher in recovering areas, especially in the driest and warmest months. Leaf transpiration, stomatal conductance, and Ci were not affected by the OTC or the land use history. The photosynthetic capacity, maximum photosynthetic capacity, and carboxylation rate of RuBisCO increased in response to warming but only in the area with no anthropogenic intervention. These results suggest that species will respond differently to warming depending on the history of páramo use, and therefore not all páramo communities will respond equally to climate change. In disturbed sites with altered soil conditions, plants could have a lower breadth of physiological response to warming.

## 1. Introduction

Climate change is affecting all ecosystems, and it is projected that, by 2030, the increasing global temperature will reach the 2 °C thresholds, relative to the pre-industrial period. [1]. Variations in precipitation, depending on region, temperature increases, increases in evapotranspiration, decreases in the soil water column [1], and loss of glaciers [2] have been observed. Warming has been reported to be much more severe in high-elevation regions, inducing cryosphere melting and intensification of the water cycle, among other effects [3,4,5]. In the Andes, a similar pattern has been observed, where the temperature has increased more at high elevations in páramo ecosystems because of the increase in latent heat coming from the Pacific Ocean [6]. These changes are projected to have a strong impact on the ecophysiology and development of plant species [7], as well as on their distribution [8]. The páramo is a high mountain tropical ecosystem, located above the tree line, characterized by a cold and humid climate, with large temperature variations throughout the day that can range from temperatures below zero at dawn to temperatures close to 22 °C at noon [9,10]. Global warming is predicted to severely affect these ecosystems, where a temperature increase of 1.5 to 3.5 °C is projected by the end of this century [11]. This warming, together with the strong anthropogenic pressure of transformations towards agricultural and livestock uses, can alter the groundwater level, and its nutritional and biotic conditions, affecting the ecophysiology of plants [12,13,14].Vegetation in the Andes is distributed in the form of islands at the top of the mountains and is characterized by a high rate of speciation, rate of endemism, and biodiversity [15,16]. This diversity is threatened by the ascent of species from lower elevations, which could displace páramo species, reducing their distribution area, as occurs in non-tropical alpine plants [17]. It has been proposed that the vegetation of high tropical mountains is very sensitive to climate change [15]; however, many of these models do not take into account the physiology of species and may underestimate their ability to acclimatize.

The photosynthetic adjustments of páramo plants to warming are not fully understood in terms of the carbon economy, and the risks or vulnerability of species in the face of increases in temperature and variability in precipitation have not been accurately determined. Current knowledge on how warming could affect the photosynthetic performance of high mountain plants comes from several studies on temperature and, alpine ecosystems; there are very few studies on subtropical and tropical mountain ecosystems [18,19,20,21,22]. Discrepancies could be related to co-limitation by temperature and water under a climate change scenario. The decrease in water reduces morphological traits such as leaf size, and leaf mass per area, and physiological traits such as maximum rate of photosynthesis, electron transport rate, stomatal conductance, and leaf water potential [6,23,24].

Responses are related to the rapid and sensitive adjustment of photosynthesis [21,25], such as greater stability of membrane integrity [26], increased ETR, and increases in Vcmax to maintain optimal photosynthetic capacity [27]. Mechanisms for acclimatization are involved in these responses, such as limitation in electron transport, expression of heat shock and chaperone proteins, and lower respiration rates [26,28,29]. or that breathing continues with optimal behavior related to maintaining long-term photosynthetic capacity [30].

One of the few studies that evaluated the response of páramo species to in situ warming using open top chambers (OTC) showed that, after three years of warming, there were no changes in photosynthetic activity, respiration, or in the net carbon exchange of the ecosystem [22]. The only few changes observed were in the structure of the vegetation, suggesting that relatively healthy páramos will resist temperature increases, but that the long-term effect depends on changes in the vegetation and initial conditions of the plant community.

The possible differential response of páramos from different sites and the possibility that land use history could affect plant response to warming led to evaluating a design of experiment that included, in addition to a passive warming treatment with OTC, the use of areas with different land use history. We hypothesized that photosynthetic capacity and photosynthetic regulatory traits such as Vcmax, gs, and ETR, may increase with OTC warming and that responses could depend on sampling dates (period) and areas with different land use history. This study evaluated photosynthetic traits after one year of warming using OTC, which determined the resilience of the responsiveness of páramo plants to the combined effect of new climatic conditions and land use given that plants that develop in these areas may have predispositions to respond to temperature increase. Therefore, these sites are included to capture these variations. The objectives were: (1) To evaluate the photosynthetic responses of six representative species in the páramo ecosystem. (2) To compare the photosynthetic response to warming in páramo plants growing in previously intervened areas and conserved areas.

## 2. Results

### 2.1. Climate

**Figure 1:**
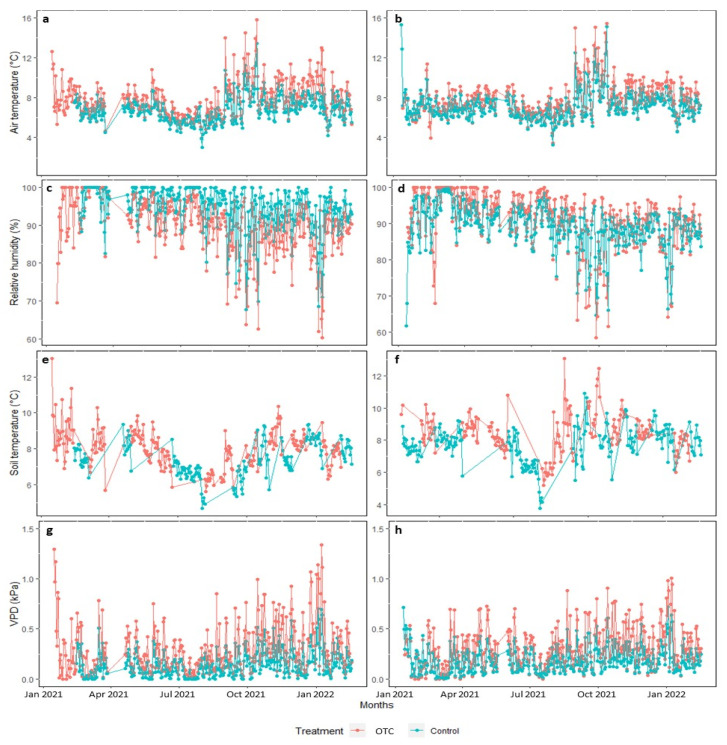
Climatic parameters throughout the experiment, daily mean values of air temperature (°C), relative humidity (%), soil temperature (°C), and pressure deficit of environmental value-DPV (kPa) in the two study areas. (**a**,**c**,**e**,**g**) conserved area and (**b**,**d**,**f**,**h**) intervened area, subjected to passive warming in the OTC treatment (red) and without passive warming in the control treatment (blue).

The OTC chambers increased the temperatures of the air and soil differently in the conserved and intervened areas (*p* < 0.05). On average, in the conserved area, the OTC treatment generated an increase of 1.5 °C in ambient temperature and 1.2 °C in soil temperature; while in the intervened area, the increase was 1.1 °C and 0.9 °C, respectively (*p* < 0.05). The relative air humidity and volumetric soil water content decreased within the OTCs in the conserved area, and the vapor pressure deficit (VPD) increased (*p* < 0.05). In the intervened area, the environmental temperature, the soil temperature, and the volumetric content of soil water increased in the OTC with respect to the control treatment, but the VPD decreased, and the relative humidity did not change (Figure 1). The OTC increased the maximum ambient and soil temperatures and reduced the minimum relative humidity of the air (*p* < 0.05).

### 2.2. Chlorophyll a Fluorescence

**Figure 2:**
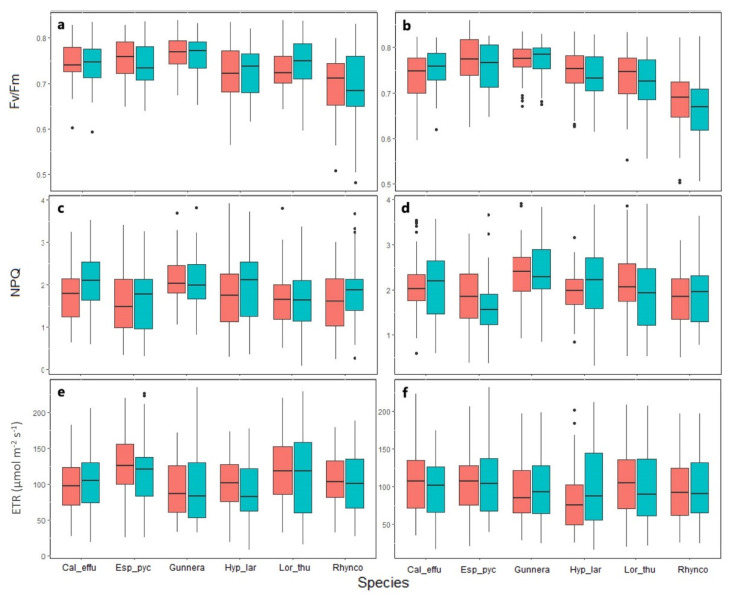
Chlorophyll a fluorescence parameters in páramo plants in two study areas. Conserved area (**left**) and intervened area (**right**), subjected to passive warming in the OTC treatment (red) and control without OTC (blue). Pooled data for all seasons, *n* = 58, *p* > 0.05. Cal_effu = *Calamagrostis effusa*, Esp_pyc = *Espeletia pycnophylla*, Gunnera = *Gunnera magellanica*, Hyp_lar = *Hypericum laricifolium*, Lor_thu = *Loricaria thuyoides*, Rhynco = *Rhynchospora macrochaeta*. Fv/Fm Maximum quantum efficiency of photosystem II (**a**,**b**), NPQ non-photochemical quenching (**c**,**d**), and ETR Electron transport rate (**e**,**f**). The black points are outliers.

No statistically significant differences were obtained in the Fv/Fm between species (Figure 2) and between OTC and control in any of the two areas (Figure 2a,b). Values differed between sampling dates (GLMM X5 = 261.07: *p* < 0.05) (Figure S1), with values slightly higher in August and January (0.75 and 0.76). *G. magellanica* had the highest Fv/Fm values, with an average of 0.77 and *R. macrochaeta* presented the lowest values (0.69).

NPQ was the only chlorophyll fluorescence parameter that showed significant differences between study areas (GLMM X2 = 2.154: *p* < 0.05) (Figure 2c,d). The NPQ values were higher in the plants from the intervented area, with an average of 2.04; while the plants from the conserved area presented an average of 1.81. Throughout the season (Figure S2), significant differences were observed, especially in the plants from the conserved area, where the heat dissipation was greater in the month of February (average = 2.14). Although lower, significant dissipation was obtained in June (average = 1.53). Between species, differences were found for *E. pycnophylla* and *R. macrochaeta*, which obtained lower NPQ values with an average of 1.67 and 1.80, respectively. As seen with Fv/Fm, no differences were observed between the OTC treatments and the controls for the species in the two study areas (Figure 2c,d).

One of the more fluctuating parameters was the ETR, which, despite not presenting differences between the study areas (Figure 2e,f), did exhibit significant fluctuations among sampling periods (Figure S3). For most of the species, lower values were obtained in August 2021 (dry period, with low average temperatures), and higher ones were seen in January 2022 (dry period). *E. pycnophylla* achieved higher ETR values than other species, with an average of 114.31 µmol m^−2^s^−1^ (Figure 2e,f). For this variable, there were also no differences between the OTC and control treatments.

### 2.3. Gas Exchange Variables

**Figure 3:**
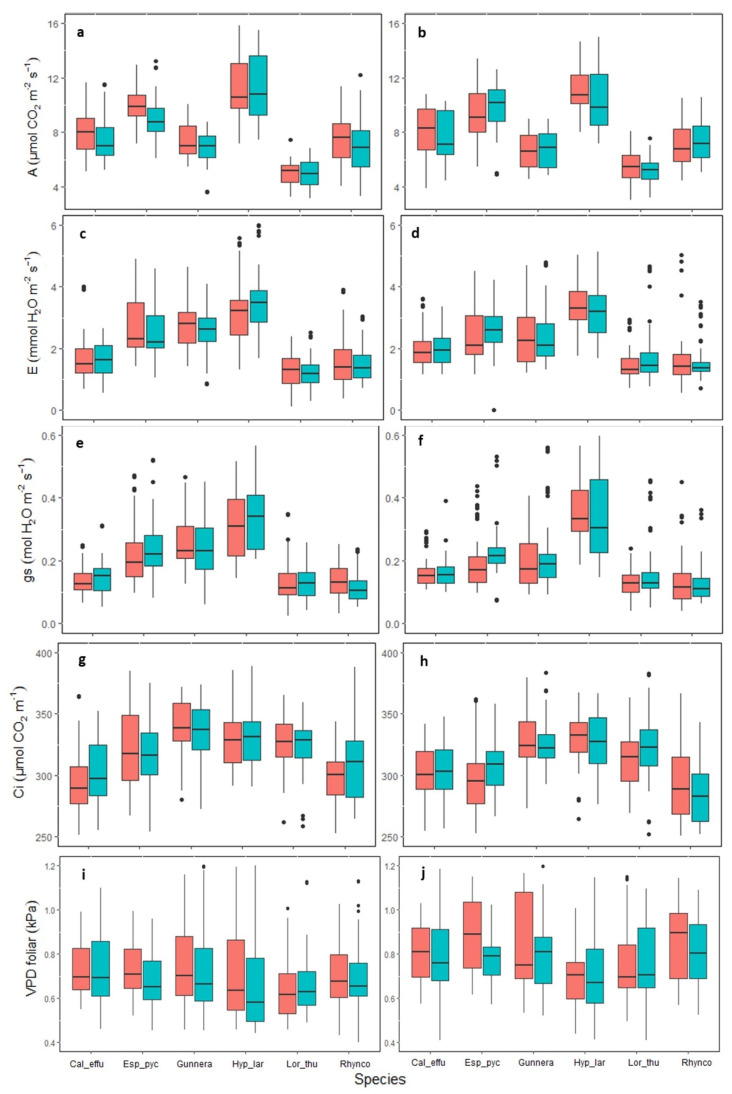
Gas exchange parameters in páramo plants in the conserved area (left) and intervened area (right), subjected to passive warming in the OTC treatment (red) and plots without passive warming in the control treatment (blue). Pooled data for all seasons, n = 75, *p* > 0.05. Cal_effu = *Calamagrostis effusa*, Esp_pyc = *Espeletia pycnophylla*, Gunnera = *Gunnera magellanica*, Hyp_lar = *Hypericum laricifolium*, Lor_thu = *Loricaria thuyoides*, Rhynco = *Rhynchospora macrochaeta*. Net photosynthesis (**a**,**b**), Transpiration (**c**,**d**), Stomatal conductance (**e**,**f**), Intercellular CO_2_ concentration (**g**,**h**), and Leaf vapor pressure deficit (**i**,**j**). The black points are outliers.

The photosynthetic rate (A) values differed among species. *H. laricifolium* presented the highest value with an average of 11.5 µmol of CO_2_ m^−2^s^−1^, while the lowest value was in *L. thuyoides* with an average of 4.8 µmol of CO_2_ m^−2^s^−1^ (Figure 3). Similarly, a significant effect of warming was found in A, being greater in the OTC, but this behavior was only observed in the species of the conserved area (GLMM X2 = 3.1278: *p* < 0.05) (Figure 3a). There were differences between the sampling dates (GLMM, X5 = 261.21: *p* < 0.05), where higher photosynthesis values occurred in June 2021 and January 2022 (dry seasons), and lower values were seen in December 2020 (season of transition from wet to dry) (Figure S4).

Leaf transpiration (E) was not affected by the OTC or the land use history. The species with the highest average transpiration was *H. laricifolium* with 3.5 µmoles of H_2_O m^−2^s^−1^, while the lowest values were in L. thuyoides with 1.8 µmoles of H_2_O m^−2^s^−1^ (Figure 3c,d). Statistically significant differences were found between the samplings of February 2021, June 2021, August 2021, and January 2022 (GLMM, X5 = 311.50: *p* < 0.05). Dry seasons generated the greatest variability (Figure S5).

Stomatal conductance gs was not affected by the OTCs nor the land use history (Figure 3e,f). The species that presented the highest values was *H. laricifolium* with an average of 0.35 mol H_2_O mm^−2^s^−1^, while *L. thuyoides* had the lowest average with 0.1 mol H_2_O mm^−2^s^−1^. Additionally, statistically significant differences were found among samplings from December 2022, August 2021, and January 2022 (GLMM, X5 = 383.54: *p* < 0.05), with the highest values in the dry season (August), and the lowest values in the wet season (December) (Figure S6).

Ci was not affected by the OTC or the land use history (Figure 3g,h). The species that presented the highest values was *H. laricifolium* with an average of 345 µmol CO_2_ m^−1^. Statistically significant differences were seen between samplings of February 2021, June 2021, August 2021, and January 2022 (GLMM, X5 = 412.77: *p* < 0.05), corresponding to the dry seasons (Figure S7).

In general, statistically significant differences were found among species, especially *E. pycnophylla*, *H. laricifolium*, *L. thuyoides,* and *G. magellanica*. Notably, *E. pycnophylla* and *H. laricifolium* showed a higher photosynthetic performance. On the other hand, *L. thuyoides* and *G. magellanica* had lower values of A with 6 µmol of CO_2_ m^−2^ s^−1^.

### 2.4. Response of Photosynthesis to Light (An/PPFD) and to CO_2_ Concentration (An/Ci)

The light response curves of the species in the two study areas after one year of passive warming showed linear responses with increasing PPFD and subsequent stabilization of the curve at saturating PPFD levels (Figure S8). After one year of warming, the maximum photosynthetic rate (Amax) presented differences between OTC and control in the conserved area (GLMM X_1_ = 8.03: *p* < 0.05) increasing with warming; as well as between species (GLMM X_1_ = 144.9: *p* < 0.05) where *H. laricifolium* and *E. pycnophylla* presented maximum rates of carbon assimilation greater among 8 and 12 μmol CO_2_ m^−2^ s^−1^, in contrast to *G. magellanica* and *L. thuyoides*, with Amax values less among 5 and 7 μmol CO_2_ m^−2^ s^−1^ (Table 1). The LCP light compensation points (Table S1) ranged from 7 to 70 μmol photons m^−2^ s^−1^in the conserved area, with the lowest average for *G. magellanica* (12 μmol photons m^−2^ s^−1^), and *H. laricifolium* with the highest average (56 μmol photons m^−2^ s^−1^). In the intervened area, the LCP values were lower, in the range of 9 to 45 μmol photons m^−2^ s^−1^. *R. macrochaeta* had the lowest compensation point (9.8 μmol photons m^−2^ s^−1^), and *L. thuyoides* had the highest average (41.5 μmol photons m^−2^ s^−1^). LSP light saturation points (Table S1) were higher in the species from the conserved area, ranging from 200 μmol photons m^−2^ s^−1^ to 900 μmol photons m^−2^ s^−1^ with a slight increase under the OTC treatment. In the intervened area, the average values are lower, between 160 μmol photons m^−2^ s^−1^ to 560 μmol photons m^−2^ s^−1^. *H. laricifolium* had the highest LSP, and *G. magellanica* had the lowest.

Dark respiration (Rd) presented values between 0.3 and 2.0 μmol CO_2_ m^−2^ s^−1^ in the conserved area and between 0.4 and 1.5 μmol CO_2_ m^−2^ s^−1^ in the intervened area. *H. laricifolium* presented higher respiration rates than the other species in both areas, and *R. macrochaeta* had the lowest rates of Rd. There were significant differences between species, such as *H. laricifolium* and *L. thuyoides*, with an Rd greater than 1 μmol CO_2_ m^−2^ s^−1^, while the average for *R. macrochaeta* was 0.5 μmol CO_2_ m^−2^ s^−1^. For the study areas, the average Rd in the conserved site was major than in the intervened area (Table 1).

The apparent quantum yield was similar between all species in the two areas, with no differences caused by the OTC treatment. The values remained constant in a range of 0.03 to 0.04 μmol (CO_2_) μmol (photon)^−1^.

The maximum rate of carboxylation of RuBisCO (Vcmax_app) had differences between OTC and control (GLMM X_1_ = 6.36: *p* < 0.05); areas (GLMM X_1_ = 8.45: *p* < 0.05); species (GLMM X_5_ = 21.15: *p* < 0.05) and sampling (season of the year) (GLMM X_5_ = 97.16: *p* < 0.05) (Table 1). The initial sampling values indicated that all species from the conserved area had higher RuBisCO carboxylation rates than the species from the intervened area; however, for the wet season (April 2021), at four months of experimentation, the Vcmax_app rates increased above 60 μmol m^−2^ s^−1^for the conserved area and 30 μmol m^−2^ s^−1^ for the intervened area. The following months of the dry season (August and January) saw Vcmax_app decrease, which was constant after eight months of passive warming. *L. thuyoides* had the lowest rates of Vcmax_app in the two areas and seasons.

**Figure 4:**
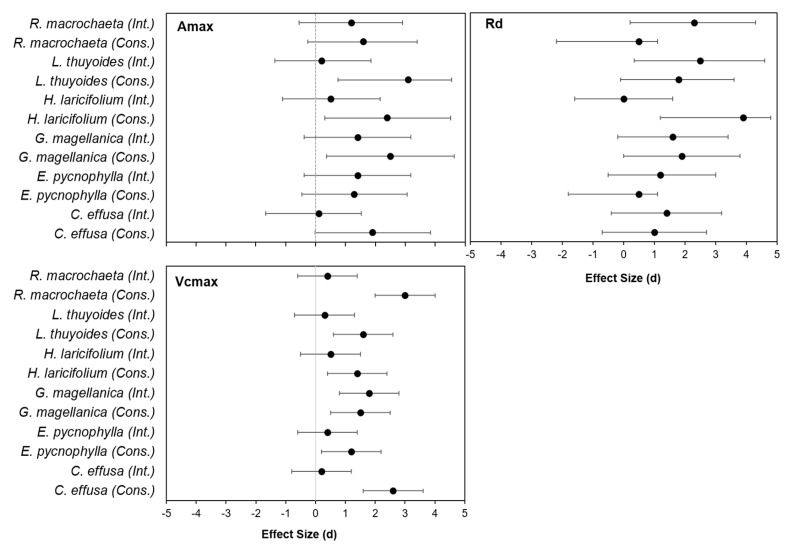
Size of the effect of the OTC treatment on the parameters Amax, Rd and Vcmax_app, derived from the response curves to light and response curves to CO_2_ after one year of passive warming in the species *E. pycnophylla*, *H. laricifolium*, *L. thuyoides*, *R. macrochaeta*, *C. effusa* and *G. magellanica* from the conserved and intervened areas.

The effect size analysis showed that, after one year of passive warming using OTC chambers, *E. pycnophylla*, *G. magellanica,* and *H. laricifolium* increased their photosynthetic performance in response to warming (Figure 4) but only in the conserved area as indicated by the large effect size (d greater than 0.8) (Figure 4).

Respiration rates also increased in response to warming for the species *L. thuyoides* from the intervened area (d = 2), for *R. macrochaeta* from the intervened area (d = 2.3), and for *H. laricifolium* (d = 3.9) from the conserved area.

The maximum carboxylation efficiency (Vcmax_app) also increased in response to warming especially in the conserved area, with the six species having a large effect size that was significant (d greater than 0.8). In the intervened area, *G. magellanica* was the only species with a significant effect size that was also large (d = 1.8) (Figure 4).

## 3. Discussion

The one-year passive warming experiment with OTC chambers increased daytime temperatures compared to controls, as has occurred in other studies and ecosystems [22,25,31,32]. In the microenvironment, the relative humidity and the volumetric water content of the soil decreased, possibly due to an increase in evapotranspiration [22,32] since the VPD of the OTC treatment was significantly higher (Figure 1g). The increase in soil temperature greater than 1 °C was probably the cause of the reduction in soil water content, this can affect plants by decreasing their productivity and photosynthetic performance [33,34,35], however, this does not occur in this study, possibly because the availability of water in the páramo ecosystem was not limiting, since it has good hydraulic conductivity and its plants have a high water retention capacity [12].

Some research has reported that a decrease in soil moisture induced by warming could be the main reason for the decrease in photosynthesis [36,37]. In this study, it was found that the soil temperature increased in the OTC treatment by 1.2 °C, while soil moisture decreased. Although these microclimate alterations in páramo endemic species that are adapted to low temperatures could condition photosynthetic performance and the efficient use of resources in the long term, the one-year data indicated that there was no immediate, negative effect on photosynthetic performance.

Parameters E, gs, and Ci did not show a differential response to the OTC treatment when compared to the control treatment; however, there were differences between species. Except for *C. effusa* and *R. macrochaeta*, both graminoids, contrasting differences were found among the other studied species, indicating that the species are differentiating factors in the responses of photosynthesis and gas exchange variables to warming, as found by Shen et al. [37] and Zhou et al. [25]. On the other hand, Elmendorf et al. [38] reported that the response to long-term passive warming in the tundra was the opposite in grasses, sedges, and rushes. While Liang et al. [39] found that the effects of warming on photosynthesis were greater in grasses than in herbs, although in the present study we cannot statistically different among growth forms.

The neutral effect of OTC on the instantaneous gas exchange variables was within the different trends reported in the response of photosynthesis to warming. This trend may be related to the C3 photosynthetic type characteristic of the páramo species. It has been found that C4 species may benefit more than C3 from warming [39]. On the contrary, Shen et al. [37] found that the net photosynthesis, stomatal conductance, intercellular CO_2_ concentration, and transpiration rate of *Leymus secalimus* were significantly decreased by passive warming. Lasso et al. [22] did not find evidence that passive warming for a period of three years generates changes in the processes of the páramo ecosystem, including photosynthesis, respiration, and productivity in the páramos of Sumapaz and Matarredonda, Colombia. Thus, evidence indicates that species tend to have different responses to warming and that reported physiological adjustments to increased temperature include reduced respiration rates [40], higher rates of carbon assimilation [41] and changes in optimum thermal photosynthesis in response to warming [42,43].

It was expected that páramo species living in conditions limited by low temperatures might respond more strongly to warming [42,44]; however, the present study showed that there was an increase in photosynthetic capacity (A, Amax and Vcmax_app), as discussed below, but not in other instantaneous variables. In páramo plants, no effects on instantaneous measures of gas exchange were observed for the one-year exposure time to the passive heating treatment, although a rapid response was found in some of the species at 8 and 12 months of the experiment (Table 1). This may be a limitation since short duration experiments may have variable responses and patterns that are difficult to estimate. However, Dusenge et al. [6] stated that physiological changes can occur in terms of months to a year and that annual monitoring may miss the variability of these responses, with processes tending towards acclimation of photosynthesis to passive heating. Although our temperature increase values are within the heating values reported in most experiments using a passive heating design with open chambers such as ours [38].

No differences were observed in the Fv/Fm of the species between the areas or treatments, which means that the plants did not present photoinhibition [45], except for *R. macrochaetta*, which presented values of 0.69, outside the range for a healthy species, with an interval between 0.74–0.85 [46]. Some results reported by Shen et al. [37] showed that passive warming caused photoinhibition in the alpine species *Leymus secalimus* and induced decreased PSII efficiency with low values of Fv/Fm and qP but increased NPQ.

Here, the NPQ presented differences between the areas, being higher in the intervened area than in the conserved area, indicating the important role that NPQ plays as a photoprotection strategy for the species of the intervened area since heat dissipation can alleviate possible stresses oxidative [47]. In this strategy, the consumption of electron equivalents of the Calvin cycle is reduced, which increases the demand for energy dissipation within photosystem II (PSII) and alternative electron sinks, all to regulate the decrease in the photosynthetic capacity and stomatal conductance [48,49,50,51]. The intervened area has microclimatic characteristics similar to the conserved area (86% RH and average air temperature of 10 °C), and a history of human intervention by quarry mining. There is probably some type of stress in plants without affecting the stability of the photosynthetic apparatus or the PSII reaction centers [52,53]. In addition, it has been reported that photosynthetic activity does not decrease in disturbed environments [54], however the adverse effects to which the plants were subjected in the intervened area can serve as drivers to improve the resistance, acclimatization, and adaptability of plants, allowing them to survive in a fluctuating environment [55] because specialization for extreme environments can potentiate characteristics, such as against temperature [56].

On the other hand, no significant differences were found in the photochemical performance of the species as measured through Fv/Fm, ETR and NPQ in response to passive warming (Figure 2). This can be attributed to the fact that plants in the Andes are prepared to deal with high irradiances and diurnal temperature variation. This was confirmed by Bravo et al. (2007) when evaluating two ecotypes of *Colobanthus quitensis* from Antarctica and the Andes, finding high resistance in the Andean ecotype to strong irradiances and high temperatures. In addition, studies such as the one by Magaña et al. [53] have shown that plants in high mountain ecosystems have a highly efficient capacity under combined drought and heat stress, indicating a good adaptive response to warming.

Since photosynthesis is directly regulated by climatic factors such as temperature, VPD and by limitations in these conditions, these factors affect ecosystem processes such as productivity, carbon, and nutrient flow. Recording the photosynthetic responses of páramo plants to temperature increases is essential to determine the degree of sensitivity of these species for future management and conservation. In this study, significant increases were found in the maximum photosynthetic capacity (Amax), in the apparent carboxylation rate of the RuBisCO enzyme (Vcmax_app) (Table 1, Figure 3a), when compared to temperature increases in the conserved area. This positive response of the species in a short time could indicate a possible dynamic acclimatization that could lead to improving the fitness of these species in the face of climate change [57,58], an analysis that needs to be verified over time.

These traits also varied with the measurement season, the species, and the area, being higher in *H. laricifolium* and *E. pycnophylla* in seasons with higher temperatures (Figure 2 and Figure 3). The results indicated a differential photosynthetic behavior in the páramo species, where it was found that *H. laricifolium* and *E. pycnophylla* had higher photosynthetic attributes, which, under a climate warming scenario, could possibly be more favored since plants have the ability to acclimatize and adapt to changing climatic conditions and tend to do so in a way that maintains or enhances carbon gain [57,59]. In addition, they would benefit from higher temperatures, which suggests that the structure of the páramo community could change with climate warming, favoring some species over others.

The significant differences in terms of areas (Table 1) showed that Vcmax_app was higher in all species from the conserved area so the background of the disturbance had negative repercussions on the photosynthetic performance of páramo plants. Possibly the disturbances that alter physical and chemical edaphic conditions can indirectly influence the photosynthetic performance of these species. Zhou et al. [60] found similar results in *Elymus nutans* and *Potentilla anserina* from the Tibetan tundra, where Vcmax_app and Jmax increased with increasing temperature. This increase could be related to changes in nitrogen redistribution and the activity of photosynthetic enzymes in leaves [61].

The effect size analysis in this study (Figure 4) corroborated the differential behavior between species in their photosynthetic characteristics, an aspect that constitutes a baseline to continue exploring the effect of long-term passive warming. Additionally, the Amax, the rate of dark respiration, and the limitation of photosynthesis such as Vcmax_app, of the species (Table 1) showed different values in individuals that grew in the conserved area with respect to the intervened one; also, they depended on the sampling season, aspects that may interact with responses to warming. These results showed that páramo species have high plasticity and competitiveness in their response to future increases in temperature and that the responses can be different between species, generating compensation between members of the plant community. On the other hand, the history of land use and season can be decisive for the response of plants to climate change.

## 4. Materials and Methods

### 4.1. Study Area

This study was carried out in the páramo located on the northeast slope of the Galeras volcano in the Galeras Flora and Fauna Sanctuary (SFFG) (1°13′15.6′′ N and 77°21′32.4′′ W) in the Department of Nariño, Colombia. With an average annual temperature of 13.3 °C and a relative humidity that varies between 60% and 88% [62].

### 4.2. Plant Material and Experimental Design

Six species with different growth forms were chosen, both in the conserved and intervened areas: shrubs (*Loricaria thuyoides* and *Hypericum laricifolium*), caulescent rosette (*Espeletia pycnophylla*), herbs (*Gunnera magellanica*) and graminoids in tillers (*Rhynchospora macrochaeta* and *Calamagrostis effusa*).

Two study areas were selected: The first was a conserved area (3745 masl), with no history of anthropogenic use. The second was an area with a history of land use of quarry mining, here called “intervened area” (3704 masl), but this area has been in passive recovery since 1985 after the Galeras Flora and Fauna Sanctuary was established. (SFFG).

Twenty plots were established in each area with different land use history. Ten warming plots inside OTCs and ten control plots outside the OTC located no more than 3 m apart from the OTCs. The distance between OTC chambers within each area was 100 m on average, OTCs are wind screens and sun traps, have an open hexagonal design with 6 pieces that join together at an angle of 60°, and have a height of 0.5 m, a lower base of 2.08 m and an upper base of 1.5 m. They are made with fiberglass, Sun lite HP 0.040′′ [63]. The open design was chosen since it affects natural conditions to a lesser extent, allowing less temperature fluctuations, improving the quantity and quality of light, and allowing natural levels of precipitation, humidity, and CO_2_ inside the chamber. It is also easily accessible for pollinators and herbivores, preventing the ecology of evaluated plants from being affected. The OTCs were installed in November 2020 and distributed in a targeted sampling design based on vegetation similarity.

### 4.3. Climatic Variables

An Em 50 microclimatic data collection system (Decagon Devices Inc., Pullman, WA, USA) with temperature, relative humidity, and PAR radiation sensors was installed in one OTC chamber and in one control of the two areas. The sensors were located at a height of 40 cm above the ground. Data were recorded every 20 min. Additionally, every 15 days, volumetric soil water content and soil temperature data were recorded at a depth of 10 cm using TEROS 12 sensors, METER Group, and ProCheck Decagon Devices data collectors.

### 4.4. Chlorophyll a Fluorescence

Induction curves were made with a saturating light pulse (Delay: 20 s, width: 40 s and 1500 μmol m^−2^ s^−1^) in leaves that were dark-adapted for 30 min using a JUNIOR PAM fluorometer (Walz, Germany), two leaves per individual, five individuals per species and treatment were measured. After the light pulse, the leaves were exposed to a series of pulses of saturating light to obtain the maximum fluorescence yield in a light-adapted state (Fm’). From the curves, the maximum quantum efficiency of photosystem II (Fv/Fm), non-photochemical quenching NPQ = (F_m_ − F_m_’)/F_m_’ [45], and the electron transport rate (ETR) were obtained, calculated as ETR = φPSII × PPFD × 0.5 × 0.84 [64]. Where φPSII is the current quantum yield of PSII (F_m_’ − F_t_ /F_m_’) [45], PPFD is the photosynthetic photon flux density, 0.5 corresponds to the partition of absorbed photons between the PSI and PSII, and 0.84 is the absorbance of the leaf.

### 4.5. Gas exchange Variables

Measurements were taken in December 2020, February, April, June, August 2021 and January 2022 with the IRGA—LICOR 6800P (Lincoln, Nebraska, USA). The gas exchange chamber was operated under an environment temperature 12 °C, 65% relative humidity, CO_2_ concentration of 400 µmol m^−2^ s ^−1^, 1500 µmol of photons m^−2^ s^−1^, 500 µmol s^−1^; flow, and an average foliar vapor pressure deficit of 0.76 kPa. Measurements were taken on consecutive days, between 8:00 a.m. and 2:00 p.m., on three leaves from five individuals of each species in OTC and in the control treatment for Net photosynthesis (A), transpiration (E), intercellular CO_2_ concentration (Ci), stomatal conductance (gs), leaf temperature (Tleaf) and vapor pressure deficit (VPD leaf) [65,66].

The response of photosynthesis to light was evaluated by constructing light curves (here called: An/PPFD) using three individuals per species in each treatment in December 2020, April 2021, August 2021 and January 2022, for 6 to 9 consecutive days between 8:00 a.m. and 2:00 p.m. under stable gas exchange chamber conditions (temperature of 12 °C, relative humidity of 65%, CO_2_ concentration of 400 µmol m^−2^ s^−1^ and flow of 500 µmol s^−1^). The net photosynthetic rate (An) was determined at different PPFD levels (2000, 1800, 1500, 1200, 900, 600, 300, 150, 50, 0 µmol photons m^−2^ s^−1^) [65,66,67]. The Microsoft Excel Solver function was used as a tool to select the best fitted An/PPFD curve. The selection was made with the minimum value of the sum of the square of the errors [68]. The model that best fit came from Kaipiainen [69], a modified Michaelis-Menten rectangular hyperbolic model.

Photosynthesis responses to CO_2_ (here called: An/Ci) were evaluated by determining An at different CO_2_ levels (in the order 400, 300, 150, 0, 400, 400, 600, 800, 1000, 1200 µmol CO_2_ m^−2^ s^−1^) at a light saturation of 1500 µmol photons m^−2^ s^−1^ under stable gas exchange chamber conditions (temperature 12 °C, 65% relative humidity, and 500 µmol s^−1^ flow). Making An/Ci curves is a time-consuming process, where there is a compromise between analyzing all plant species in all treatments and making more complete curves, with a large number of CO_2_ concentrations. For this reason, curves were made at ten different CO_2_ concentrations to shorten the measurement time until completing three curves per species per treatment and per area. Three regions were visually differentiated in each curve CO_2_ [70]. However, enough degrees of freedom was lost in the CO_2_-bound region for precise simultaneous retrieval of maximum carboxylation efficiency (Vcmax) and mesophyll CO_2_ conductance (gm), as proposed by current approaches for An/Ci analysis [71,72,73]. Consequently, the photosynthetic parameters were estimated without considering gm, that is, following the simplified procedure explained by Long and Bernacchi [70]. Therefore, the estimated Vcmax was a combination of gm-associated diffusional effects and biochemical effects associated with RuBisCO carboxylation, called the ‘apparent’ Vcmax, i.e., Vcmax_app.

### 4.6. Statistical Analysis

The data obtained for each photosynthetic trait were averaged, and the normality, homoscedasticity, and independence tests (measurements were made on the same plants during all sampling dates) were applied with the Shapiro Wilk, Levens and Durbin Watson tests, respectively, which were not significant (*p* > 0.05). Therefore, generalized linear mixed models were made with fixed effects (species, sampling date, area and treatment) and random effects (identity of the plot (Trat_id) and identity of the individual (Ind_id). The glmer function from the lm4 package was used, and the equation for the photosynthetic rate variable (An) was: MMGAn = glmer (An~Area × Treatment + Species × Treatment+Sampling (1|Trat_id/Ind_id), family = Gamma (link = “log”), data = data). This formula was applied to all variables. For all tests, an alpha = 0.05 was used. Analyses were performed with R-studio version 4.1.1.

To determine the effect of warming on photosynthetic responses to radiation and CO_2_ concentration in the two study areas (conserved and intervened), the effect size (d) was calculated using the Effect Size Calculator spreadsheet in Microsoft Excel. From the means, the number of data (n = 3) and the standard deviation of the variables were analyzed in the OTC and control treatments. The standardized effect size of Hedges and Olkin [74] was calculated, using the difference between the two means, divided by the pooled estimate of the standard deviation, and calibrating the difference between the OTC and control groups. Similarly, the confidence interval and the upper and lower confidence limits for the effect size were calculated, as explained by Hedges and Olkin [74] when 95% CI of d (effect size) did not overlap with zero, the OTC warming treatment had a significant effect size. Positive CI values indicated a positive effect from warming, and negative values indicated a negative effect. The effect was classified into 3 categories: large effect when d was ≥0.8, medium effect if d = 0.5, and small effect when d = 0.2.

## 5. Conclusions

There are few studies that approach the photosynthetic response of plants in páramo ecosystems in the context of climate change, for this reason this study determines the degree of sensitivity of the species to future global changes, which will contribute to the management, conservation or restoration processes. In conclusion, this study demonstrates that the effects of warming differ depending on the land use history. In this sense, the páramo species included in this study showed an increment of photosynthesis, A, Amax, and Vcmax app, as a consequence of warming only in the conserved areas where the anthropogenic influence has been very low. By contrast, in disturbed areas, we did not observe a clear effect of warming on the productive parameters. This fact may have relevant consequences on the structure of the páramo communities, favoring the performance of some species over others under climate change conditions and depending on their recent land use.

## Figures and Tables

**Table 1:** Parameters Amax, Rd, and Vcmax_app, derived from the response curves to light and response curves to CO_2_ in *E. pycnophylla*, *H. laricifolium*, *L. thuyoides*, *R. macrochaeta*, *C. effusa* and, *G. magellanica*, under conditions of the warming with OTC and control, monitored for one year of the experiment. Values are the sample means ± standard deviation (n = 3).

	Conserved Area								
	Dec-20		Apr-21		Aug-21		Jan-22		
Amax (μmol m^−2^ s^−1^)	OTC	Control	OTC	Control	OTC	Control	OTC	Control	Treatment Significance
*C. effusa*	10.9 ± 0.04	10.7 ± 0.1	7.9 ± 0.1	7.7 ± 0.04	8.3 ± 0.6	7.4 ± 0.4	10.4 ± 1.7	7.5 ± 0.1	*p* < 0.01
*E. pycnophylla*	17.7 ± 1.7	15.1 ± 0.9	9.3 ± 0.2	9.0 ± 0.1	13.2 ± 0.1	11.7 ± 0.3	11.9 ± 2.1	8.5 ± 2.1	
*G. magellanica*	10.3 ± 0.2	9.8 ± 0.3	7.6 ± 0.1	7.4 ± 0.1	7.1 ± 1.1	7.4 ± 0.1	8.4 ± 0.5	7.2 ± 0.02	
*H. laricifolium*	14.0 ± 0.1	13.7 ± 0.1	8.8 ± 0.01	8.7 ± 0.04	12.2 ± 1.9	9.9 ± 0.1	11.5 ± 0.3	12.5 ± 0.4	
*L. thuyoides*	13.1 ± 0.2	12.2 ± 0.03	8.5 ± 0.01	8.4 ± 0.04	6.0 ± 0.9	5.6 ± 0.7	6.0 ± 0.3	4.9 ± 0.2	
*R. macrochaeta*	11.7 ± 0.4	11.1 ± 0.1	8.2 ± 0.1	8.1 ± 0.03	9.2 ± 0.7	10.1 ± 0.3	7.8 ± 1.8	5.2 ± 0.5	
Rd (μmol CO_2_ m^−2^ s^−1^)									
*C. effusa*	0.4 ± 0.01	0.4 ± 0.1	0.7 ± 0.1	0.8 ± 0.1	0.4 ± 0.1	0.4 ± 0.1	1 ± 0.2	0.8 ± 0.04	*p* > 0.05
*E. pycnophylla*	0.6 ± 0.1	0.7 ± 0.2	0.8 ± 0.2	1.0 ± 0.3	0.7 ± 0	0.7 ± 0.02	0.7 ± 0.2	0.9 ± 0.3	
*G. magellanica*	0.9 ± 1.1	0.9 ± 0.6	0.9 ± 0.6	0.5 ± 0.04	0.4 ± 0.2	0.6 ± 0.1	1.1 ± 0.4	0.5 ± 0.02	
*H. laricifolium*	2.0 ± 0.2	2.0 ± 0.01	1.3 ± 0.3	0.8 ± 0.2	2.2 ± 0.1	1.1 ± 0.01	2.1 ± 0.03	1.5 ± 0.2	
*L. thuyoides*	0.6 ± 0.3	1.3 ± 0.3	1.5 ± 0.2	0.7 ± 0.03	1.8 ± 0.4	0.9 ± 0.3	1.2 ± 0.4	0.5 ± 0.1	
*R. macrochaeta*	0.2 ± 0.1	0.2 ± 0.04	0.5 ± 0.6	0.6 ± 0.6	0.6 ± 0.2	0.3 ± 0.1	0.4 ± 0.02	0.5 ± 0.2	
Vcmax_app (μmol m^−2^ s^−1^)									
*C. effusa*	25.4 ± 2.52	17.2 ± 1.14	69.6 ± 5.92	65.5 ± 22	24.9 ± 4.73	24.6 ± 0.93	49.4 ± 1.28	45.4 ± 0.37	*p* < 0.01
*E. pycnophylla*	20.7 ± 1.08	33.3 ± 6.37	65.6 ± 5.43	52.8 ± 0.87	42.2 ± 3.88	39.7 ± 8.3	47.3 ± 2.6	43.2 ± 2.85	
*G. magellanica*	31.0 ± 1.25	24.3 ± 0.35	84.9 ± 1.94	74.5 ± 0.49	41.6 ± 7.48	30.8 ± 8	40.1 ± 1.59	36.9 ± 1.67	
*H. laricifolium*	22.6 ± 7.23	44.4 ± 7.02	65.0 ± 1.97	43.8 ± 0.69	39.7 ± 6.81	35.3± 5.53	40.1 ± 6.9	31.6 ± 0.39	
*L. thuyoides*	28.0 ± 0.06	23.2 ± 2.62	48.6 ± 0.61	34.4 ± 0.75	42.8 ± 1.49	24.8 ± 1.41	34.3 ± 0.42	31.7 ± 1.75	
*R. macrochaeta*	26.7 ± 1.37	28.6 ± 1.46	63.1 ± 2.43	77.3 ± 3.5	47.6 ± 6.08	30.1 ± 7.2	51.4 ± 5.78	34.3 ± 2.9	
	Intervened Area								
	Dec-20		Apr-21		Aug-21		Jan-22		
Amax (μmol m^−2^ s^−1^)	OTC	Control	OTC	Control	OTC	Control	OTC	Control	Treatment significance
*C. effusa*	6.2 ± 0.1	6 ± 0.1	4 ± 0.1	3.8 ± 0.1	9.5 ± 0.8	10 ± 0.1	8.5 ± 1.8	8.6 ± 0.01	*p* > 0.05
*E. pycnophylla*	7.3 ± 0.01	7.2 ± 0.04	5.7 ± 0.1	5.6 ± 0.1	8.9 ± 1.1	5.4 ± 1.8	11.1 ± 1	12.5 ± 0.6	
*G. magellanica*	5.9 ± 0.01	5.8 ± 0.03	3.5 ± 0.1	3.1 ± 0.2	6.7 ± 0.3	4 ± 0.2	6.5 ± 0.03	6.9 ± 0.3	
*H. laricifolium*	6.9 ± 0.01	6.6 ± 0.02	5.4 ± 0.1	5.1 ± 0.1	9.4 ± 0.02	8.3 ± 0.5	12.9 ± 0.1	12.8 ± 0.2	
*L. thuyoides*	6.5 ± 0.01	6.5 ± 0.02	5 ± 0.1	4.7 ± 0.02	5.7 ± 0.02	5.4 ± 0.2	6.3 ± 0.2	6.2 ± 0.4	
*R. macrochaeta*	6.4 ± 0.01	6.3 ± 0.04	4.6 ± 0.1	4.4 ± 0.1	4.5 ± 0.4	7.7 ± 2.6	7.7 ± 0.5	9.3 ± 1.5	
Rd (μmol CO_2_ m^−2^ s^−1^)									
*C. effusa*	0.8 ± 0.1	0.4 ± 0.2	0.5 ± 0.1	0.8 ± 0.1	0.8 ± 0.1	0.4 ± 0.1	0.4 ± 0.2	0.7 ± 0.1	*p* > 0.05
*E. pycnophylla*	0.7 ± 0.03	0.9 ± 0.1	0.6 ± 0.2	0.6 ± 0.2	0.8 ± 0.1	0.8 ± 0.1	0.6 ± 0.1	0.7 ± 0	
*G. magellanica*	0.8 ± 0.1	1 ± 0.1	0.9 ± 0.1	0.9 ± 0.1	0.9 ± 0.2	0.8 ± 0.1	0.7 ± 0.1	0.9 ± 0.02	
*H. laricifolium*	1.4 ± 0.1	1.7 ± 0.04	1.5 ± 0.3	1.4 ± 0.01	1.2 ± 0.2	1.3 ± 0.1	1.5 ± 0.02	1.5 ± 0.1	
*L. thuyoides*	1 ± 0.1	1 ± 0.1	0.4 ± 0	1.3 ± 0.1	1.1 ± 0.1	1.1 ± 0.2	0.8 ± 0.2	1.2 ± 0.1	
*R. macrochaeta*	0.4 ± 0.02	0.6 ± 0.02	0.7 ± 0.2	0.6 ± 0.1	0.6 ± 0.1	0.7 ± 0.1	0.6 ± 0.04	0.5 ± 0.03	
Vcmax_app(μmol m^−2^ s^−1^)									
*C. effusa*	43.65 ± 1.75	43.9 ± 0.04	38.05 ± 6.45	38.9 ± 8.55	26.83 ± 1.79	25.34 ± 0.8	22.64 ± 1.74	22.93 ± 0.01	*p* > 0.05
*E. pycnophylla*	12.05 ± 0.65	40.3 ± 6	41.25 ± 5.95	30.5 ± 0.2	25.4 ± 1.16	22.03 ± 1.5	27.69 ± 2.89	28.71 ± 0.39	
*G. magellanica*	25.17 ± 9.2	30.95 ± 0.55	55.25 ± 4.15	35.8 ± 3.7	21.41 ± 0.42	12.01 ± 0.4	27.12 ± 1.83	24.07 ± 0.5	
*H. laricifolium*	23.7 ± 0.7	71.35 ± 0.15	48.65 ± 6.95	48.9 ± 0.9	28.4 ± 4.18	21.86 ± 0.65	33.01 ± 1.16	31.63 ± 3.17	
*L. thuyoides*	15.35 ± 1.25	16.9 ± 2.1	19.65 ± 4.25	36.6 ± 19.93	16.64 ± 0.86	16.11 ± 1.76	25.98 ± 1.75	26.74 ± 2.05	
*R. macrochaeta*	37.4 ± 6	66.6 ± 9	20.7 ± 4.8	20.25 ± 2.65	17.64 ± 1.98	21.11 ± 3.94	21.07 ± 1.76	21.84 ± 1	

## Supplementary Materials

The following supporting information can be downloaded at: https://www.mdpi.com/article/10.3390/plants11223110/s1, Figure S1: Maximum quantum efficiency of photosystem II (Fv/Fm) in páramo plants in the two study areas, conserved area and intervened area, subjected to passive warming in the OTC treatment and control plots; Figure S2: Non-photochemical quenching in páramo plants in the two study areas, conserved area and intervened area, subjected to passive warming in the OTC treatment and control plots; Figure S3: Electron transport rate in páramo plants in the two study areas, conserved area and intervened area, subjected to passive warming in the OTC treatment and control plots; Figure S4: Photosynthesis rate in páramo plants in the two study areas, conserved area and intervened area, both subjected to passive warming in the OTC treatment and without passive warming in the control plots; Figure S5: Transpiration rate in páramo plants in the two study areas, conserved area and intervened area, both subjected to passive warming in the OTC treatment and without passive warming in the control plots; Figure S6: Stomatal conductance in páramo plants in the two study areas, conserved area and intervened area, both subjected to passive warming in the OTC treatment and without passive warming in the control plots; Figure S7: Intercellular CO_2_ concentration in páramo plants in the two study areas, conserved area and intervened area, both subjected to passive warming in the OTC treatment and without passive warming in the control plots; Figure S8: Light response curves in *E. pycnophylla*, *H. laricifolium*, *L. thuyoides*, *R. macrochaeta*, *C. effusa* and *G. magellanica* under the OTC treatment and control after 12 months of treatment, both in conserved and intervened areas. Table S1: Parameters LCP light compensation point, LSP light saturation point, and apparent quantum efficiency ΦCO_2_, derived from light response curves in *E. pycnophylla*, *H. laricifolium*, *L. thuyoides*, *R. macrochaeta*, *C. effusa,* and *G. magellanica*, under the OTC treatment and control treatment, monitored for one year of the experiment.
