# Supplementary material for: Photosynthetic Traits of Páramo Plants Subjected to Short-Term Warming in OTC Chambers
Source: Plants (Basel). 2022 Nov 15;11(22):3110. doi: 10.3390/plants11223110 (PMC9695496; doi:10.3390/plants11223110)
Supplement: Supplementary file 1 [file plants-11-03110-s001.zip › plants-1981193-supplementary.pdf]

Supplementary materials

PLANTS

Photosynthetic traits of páramo plants subjected to short-term warming in OTC chambers

María Elena Solarte*, Yisela Solarte Erazo, Elizabeth Ramírez Cupacán, Camila Enríquez Paz, Luz Marina Melgarejo *, Eloisa Lasso, Jaume Flexas and Javier Gulias.

Correspondence: MES; msolarte65@udenar.edu.co; Tel.: 3214517719; +57;
LMM; lmmelgarejom@unal.edu.co; 3133481285; +57)

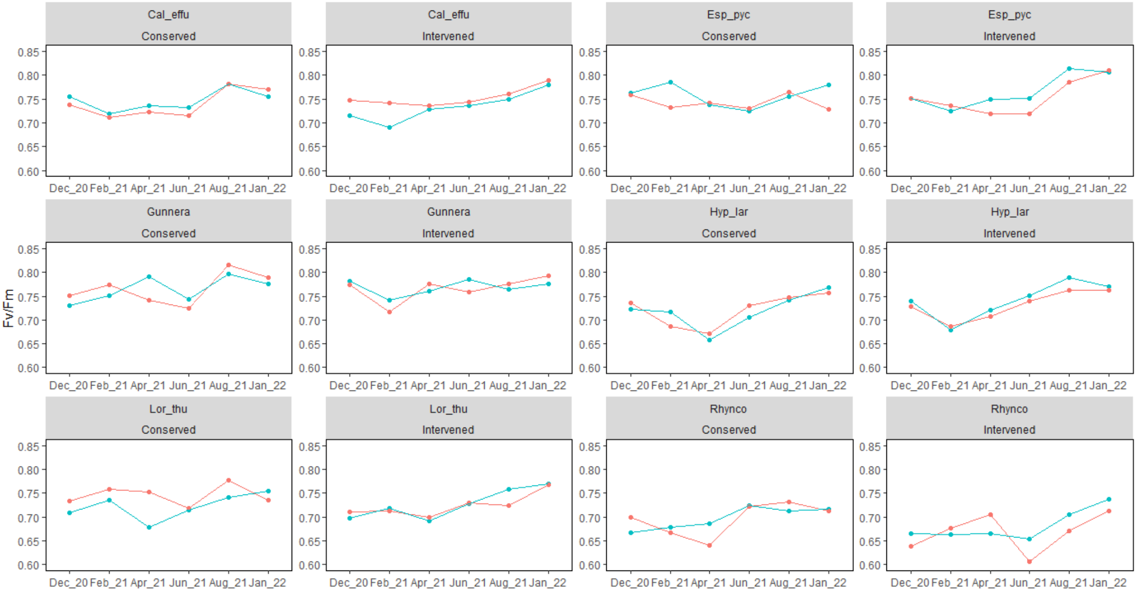

Figure S1. Maximum quantum efficiency of photosystem II (Fv/Fm) in páramo plants in the two study areas, conserved area and intervened area, subjected to passive warming in the OTC treatment (red) and control plots (blue), n = 58, p = 0.05. Cal_effu=*Calamagrostis effusa*, Esp_pyc=*Espeletia pycnophylla*, Gunnera=*Gunnera magellanica*, Hyp_lar = *Hypericum laricifolium*, Lor_thu= *Loricaria thuyoides*, Rhynco=*Rhynchospora macrochaeta*.

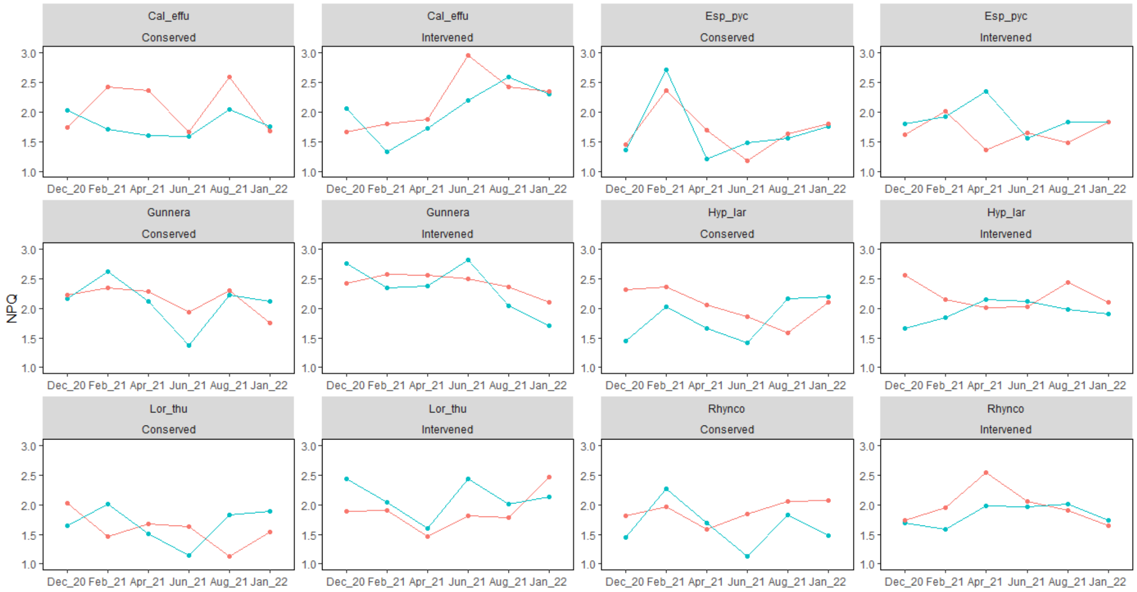

Figure S2. Non-photochemical quenching in páramo plants in the two study areas, conserved area and intervened area, subjected to passive warming in the OTC treatment (red) and control plots (blue), n = 58, p = 0.05. Cal_effu=*Calamagrostis effusa*, Esp_pyc=*Espeletia pycnophylla*, Gunnera=*Gunnera magellanica*, Hyp_lar = *Hypericum laricifolium*, Lor_thu= *Loricaria thuyoides*, Rhynco=*Rhynchospora macrochaeta*.

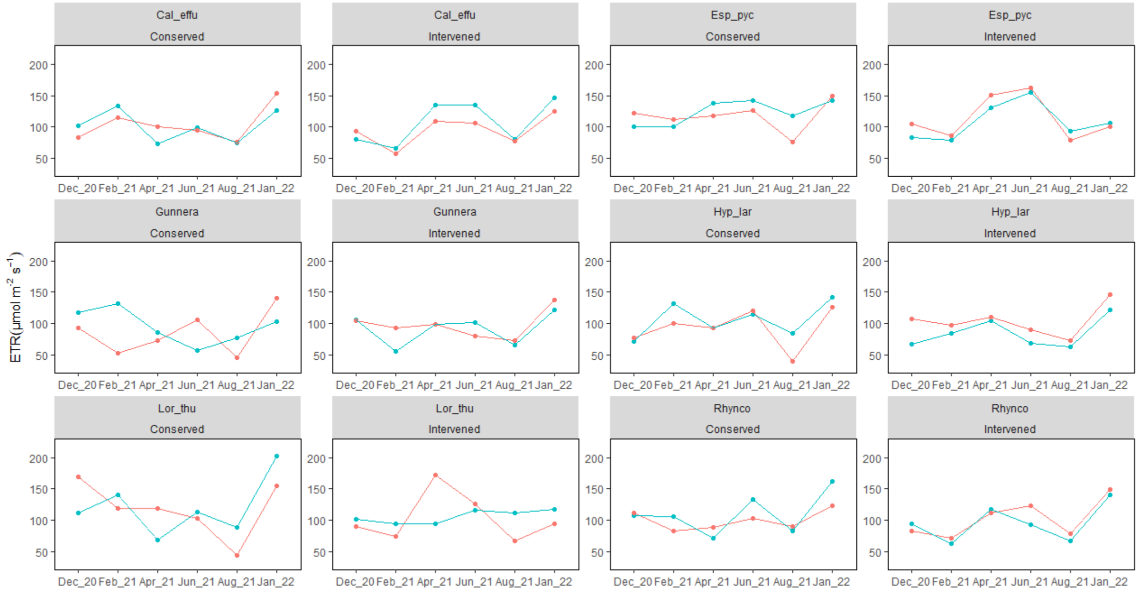

Figure S3. Electron transport rate in páramo plants in the two study areas, conserved area and intervened area, subjected to passive warming in the OTC treatment (red) and control plots (blue), n = 58, p = 0.05. Cal_effu=*Calamagrostis effusa*, Esp_pyc=*Espeletia pycnophylla*, Gunnera=*Gunnera magellanica*, Hyp_lar = *Hypericum laricifolium*, Lor_thu= *Loricaria thuyoides*, Rhynco=*Rhynchospora macrochaeta*.

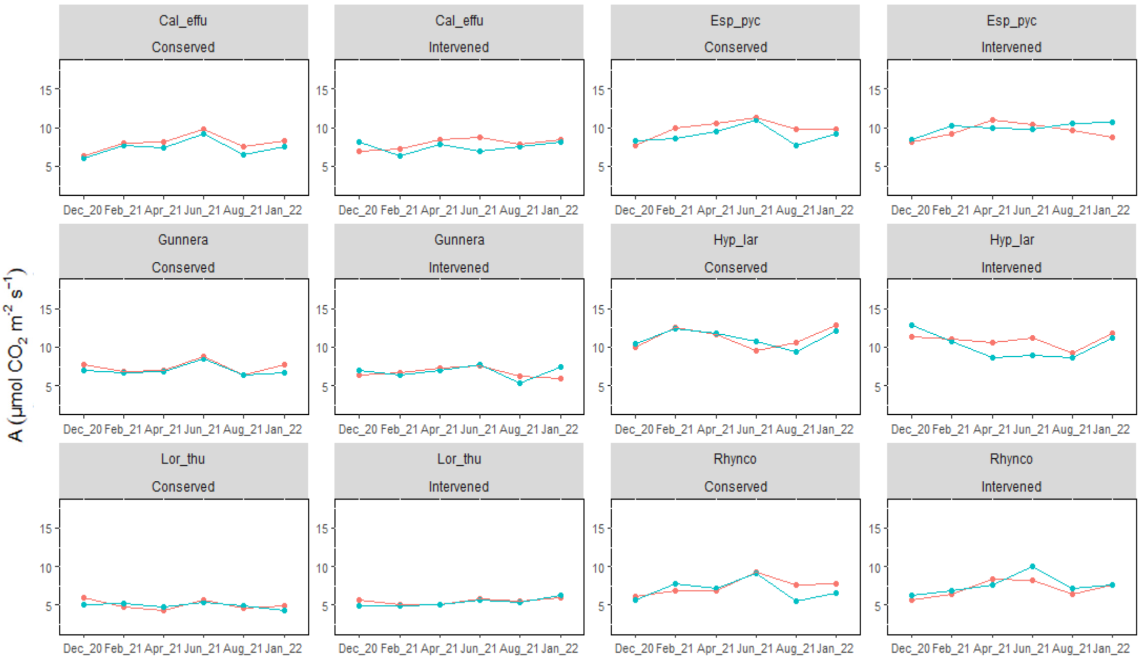

Figure S4. Photosynthesis rate in páramo plants in the two study areas, conserved area and intervened area, both subjected to passive warming in the OTC treatment (red) and without passive warming in the control plots (blue), n=75, $p>0.05$. Cal_effu=*Calamagrostis effusa*, Esp_pyc=*Espeletia pycnophylla*, Gunnera=*Gunnera magellanica*, Hyp_lar = *Hypericum laricifolium*, Lor_thu= *Loricaria thuyoides*, Rhynco=*Rhynchospora macrochaeta*.

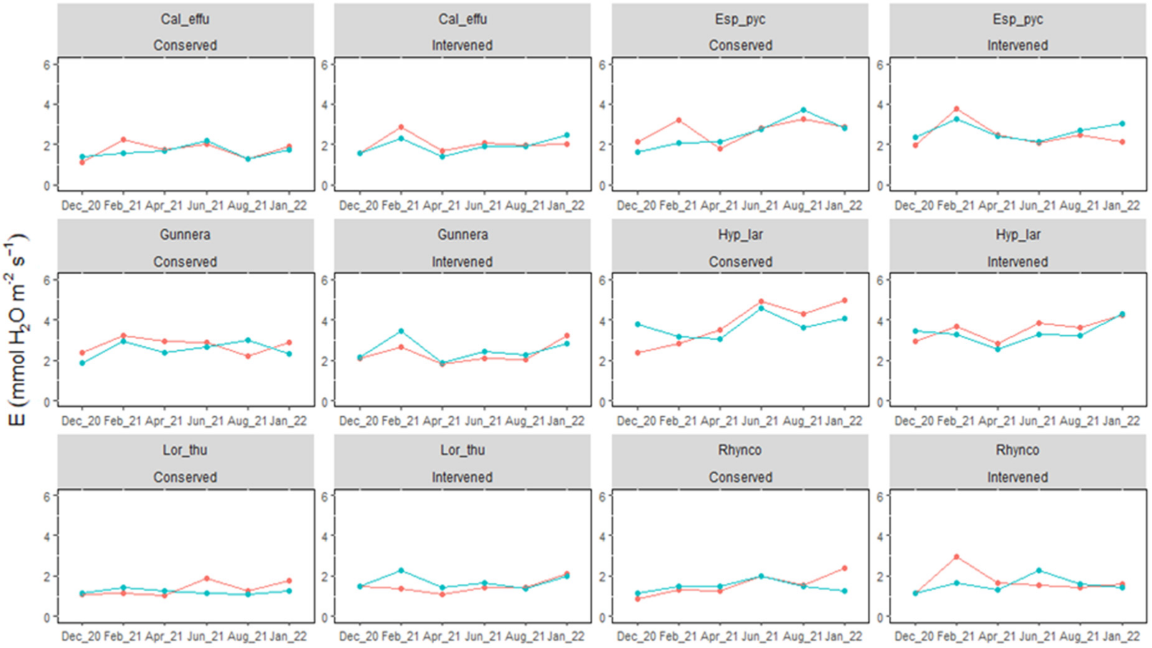

Figure S5. Transpiration rate in páramo plants in the two study areas, conserved area and intervened area, both subjected to passive warming in the OTC treatment (red) and without passive warming in the control plots (blue), n=75, $p>0.05$. Cal_effu=*Calamagrostis effusa*, Esp_pyc=*Espeletia pycnophylla*, Gunnera=*Gunnera magellanica*, Hyp_lar = *Hypericum laricifolium*, Lor_thu= *Loricaria thuyoides*, Rhynco=*Rhynchospora macrochaeta*.

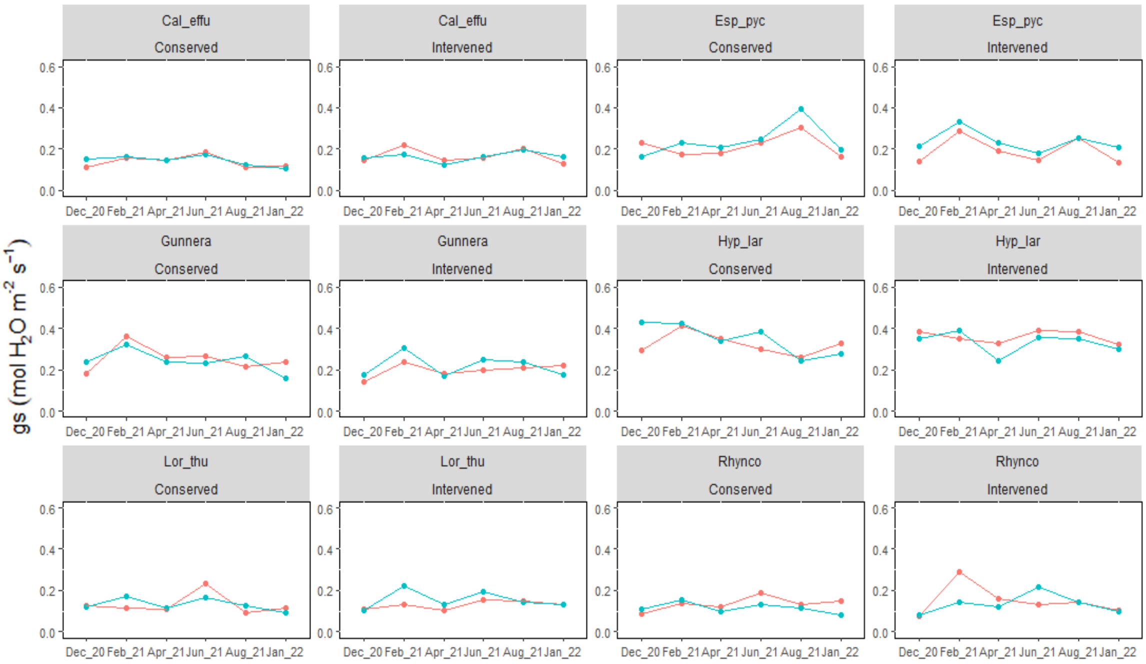

Figure S6. Stomatal conductance in páramo plants in the two study areas, conserved area and intervened area, both subjected to passive warming in the OTC treatment (red) and without passive warming in the control plots (blue), n=75, $p>0.05$. Cal_effu=*Calamagrostis effusa*, Esp_pyc=*Espeletia pycnophylla*, Gunnera=*Gunnera magellanica*, Hyp_lar = *Hypericum laricifolium*, Lor_thu= *Loricaria thuyoides*, Rhynco=*Rhynchospora macrochaeta*.

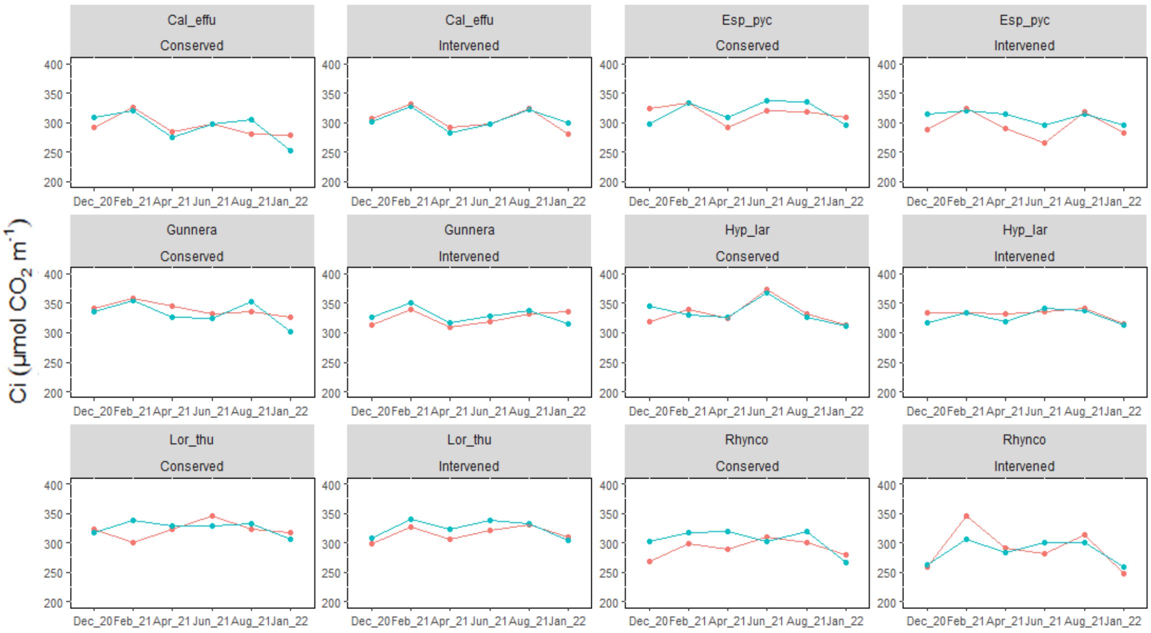

Figure S7. Intercellular CO_2 concentration in páramo plants in the two study areas, conserved area and intervened area, both subjected to passive warming in the OTC treatment (red) and without passive warming in the control plots (blue), n=75, p>0.05. Cal_effu=*Calamagrostis effusa*, Esp_pyc=*Espeletia pycnophylla*, Gunnera=*Gunnera magellanica*, Hyp_lar = *Hypericum laricifolium*, Lor_thu= *Loricaria thuyoides*, Rhynco=*Rhynchospora macrochaeta*.

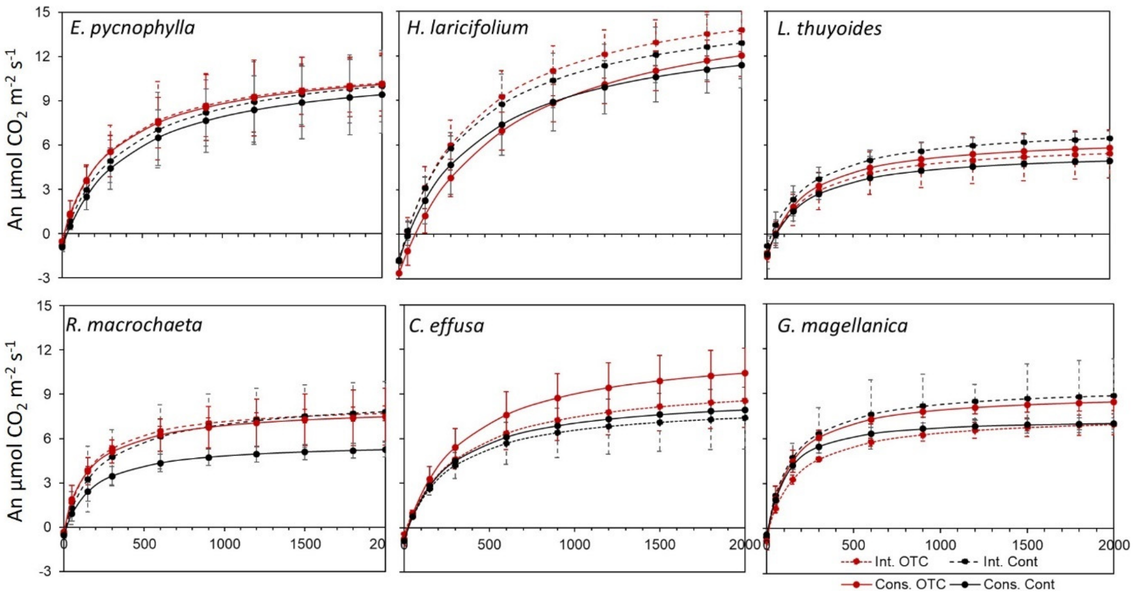

Figure S8. Light response curves in *E. pycnophylla*, *H. laricifolium*, *L. thuyoides*, *R. macrochaeta*, *C. effusa* and *G. magellanica* under the OTC treatment and control after 12 months of treatment, both in conserved and intervened areas. Int.OTC, Cons.OTC: intervened and conserved area with the OTC treatment. Int. Cont, Cons.Cont: controls without the OTC treatment of the intervened and conserved areas.

Table S1. Parameters LCP light compensation point, LSP light saturation point and apparent quantum efficiency CO_2, derived from light response curves in *E. pycnophylla*, *H. laricifolium*, *L. thuyoides*, *R. macrochaeta*, *C. effusa* and *G. magellanica*, under the OTC treatment and control treatment, monitored for one year of the experiment. Values are sample means ± standard deviation (n = 3).

	CONSERVED AREA								
	Dec-20		Apr-21		Aug-21		Jan-22		
LCP (μmol photons m^{-2} s^{-1})	OTC	Control	OTC	Control	OTC	Control	OTC	Control	Treatment Significance
C. effusa	11,7 ± 1,3	9,3 ± 1,9	27,2 ± 3,8	20,2 ± 2,6	9 ± 2,5	15 ± 2,1	19,8 ± 6,8	22,6 ± 0,2	p>0.05
E. pycnophylla	12,1 ± 1,6	17,9 ± 4,1	23,8 ± 1,5	26 ± 3,9	16,2 ± 0,6	21 ± 1,1	22,1 ± 11,2	31 ± 1,6	
G. magellanica	20 ± 13,8	15,6 ± 4	14 ± 1,6	11,3 ± 5	19,4 ± 4,4	9,2 ± 1,8	18,1 ± 8	7,3 ± 0,1	
H. laricifolium	37 ± 5,8	42,7 ± 3,5	16,9 ± 2,4	29,8 ± 0,8	50 ± 3,8	40 ± 1,3	73,9 ± 1,2	38,1 ± 10,4	
L. thuyoides	20,6 ± 12,2	34,5 ± 13,9	46,8 ± 4,2	36,2 ± 8,5	61 ± 15,5	31,3 ± 2,5	32,4 ± 13,5	28,1 ± 4,7	
R. macrochaeta	7,7 ± 0,9	6,9 ± 1,5	29,1 ± 6,3	42 ± 10,7	14,5 ± 4,9	9 ± 4,6	9,6 ± 0,1	17,8 ± 12,8	
LSP (μmol photons m^{-2} s^{-1})									
C. effusa	489,4 ± 70,6	525,7 ± 45	555,2 ± 219,8	419 ± 17,8	427,3 ± 17,6	638 ± 26,3	592,6 ± 18,5	471,9 ± 20,5	p>0.05
E. pycnophylla	434,6 ± 103,8	707,1 ± 206,2	771,4 ± 92,6	603,6 ± 118,5	852,5 ± 24,1	879,8 ± 10,3	816,8 ± 56,4	723 ± 145,8	
G. magellanica	231,9 ± 52,6	202,5 ± 53,9	277,8 ± 36,6	230,8 ± 86,1	364,2 ± 166	260,7 ± 20,6	289,8 ± 62,4	196,7 ± 13,8	
H. laricifolium	827,2 ± 25,6	898 ± 82,8	551 ± 349,7	740,1 ± 52,9	854,4 ± 18,7	941 ± 122,2	905,8 ± 56,3	709,3 ± 98,2	
L. thuyoides	410,1 ± 40,3	501,7 ± 11,5	319,9 ± 36,7	286,8 ± 87,2	704,3 ± 24,2	616,1 ± 142,6	368,5 ± 2,6	386,3 ± 34,8	
R. macrochaeta	302,9 ± 106,3	223,7 ± 64	354 ± 177,3	111,8 ± 11,8	623,7 ± 80,4	622,4 ± 97,5	452,5 ± 148,5	347,6 ± 81	

LCP (μmol photons m^{-2} s^{-1})	INTERVENED AREA								
	Dec-20		Apr-21		Aug-21		Jan-22		
	OTC	Control	OTC	Control	OTC	Control	OTC	Control	Treatment Significance
C. effusa	23 ± 2,5	13,7 ± 2,4	17,4 ± 0,8	22,5 ± 3,3	21 ± 3,2	10,7 ± 1,4	16,8 ± 1,3	20,4 ± 2,9	
E. pycnophylla	11,9 ± 1	16,3 ± 2,4	13,6 ± 5,8	13,3 ± 1	18,5 ± 1,2	17,9 ± 1,9	13,3 ± 0,6	16,9 ± 1,1	
G. magellanica	13,6 ± 1,5	13,6 ± 3,2	11,6 ± 1,3	17,4 ± 1,7	15,9 ± 2	15,1 ± 1,5	9 ± 0,6	13,6 ± 0,8	p>0.05
H. laricifolium	34,8 ± 2,7	37,8 ± 2,8	39,9 ± 6,1	36,5 ± 4	34,1 ± 1	36,2 ± 4,2	37,9 ± 3,4	39,9 ± 1,6	
L. thuyoides	49,7 ± 2,8	41,3 ± 0,8	27,2 ± 0,8	54,7 ± 1,5	43,1 ± 1,2	42,1 ± 1,8	37,7 ± 5,8	45,3 ± 0,1	
R. macrochaeta	6,9 ± 0,1	9 ± 0,4	16,3 ± 6,7	16 ± 5,6	20,1 ± 6,1	20,6 ± 7,8	11,4 ± 0,1	8,1 ± 0,1	
LSP (μmol photons m^{-2} s^{-1})									
C. effusa	445,3 ± 151,8	524,3 ± 17,5	434,6 ± 53,8	619,6 ± 3,9	739,9 ± 2,4	390,8 ± 31,1	298,1 ± 38,8	274,3 ± 18,1	
E. pycnophylla	455,8 ± 32,2	419,8 ± 66,2	352,3 ± 76,9	382,6 ± 81,2	360,8 ± 76	303,5 ± 39	328,6 ± 21,2	267,2 ± 14,6	
G. magellanica	221,9 ± 21,5	192,4 ± 18	205,5 ± 31,5	248,8 ± 13,1	284,5 ± 4,9	157,9 ± 10,7	177,9 ± 28,9	166 ± 8,6	p>0.05
H. laricifolium	564,9 ± 94,4	733,5 ± 27,2	549,6 ± 27,5	538,1 ± 105	830,6 ± 81,8	606,7 ± 39,3	438,2 ± 106	560,8 ± 43,5	
L. thuyoides	418,7 ± 11,4	364,0± 42,6	363,7 ± 55	342,2 ± 7,2	496,4 ± 38,8	428,3 ± 24,8	229,7 ± 15,9	209 ± 28,8	
R. macrochaeta	188,2 ± 34,3	267,3 ± 105,8	314,2 ± 29,4	352,8 ± 34,1	294,6 ± 52,1	354,5 ± 41,9	158 ± 28,2	169,7 ± 13,5	
